# miR-370-3p as a Novel Biomarker Promotes Breast Cancer Progression by Targeting FBLN5

**DOI:** 10.1155/2021/4649890

**Published:** 2021-08-23

**Authors:** Jiahui Mao, Lingxia Wang, Junying Wu, Yichun Wang, Huiyan Wen, Xueming Zhu, Bo Wang, Huan Yang

**Affiliations:** ^1^Department of Central Laboratory, The Affiliated Hospital of Jiangsu University, Zhenjiang, 212013 Jiangsu, China; ^2^Department of Clinical Laboratory, The Second Affiliated Hospital of Soochow University, Suzhou, 215004 Jiangsu, China; ^3^Department of Oncology, The Second Affiliated Hospital of Soochow University, Suzhou, 215004 Jiangsu, China

## Abstract

miRNAs play a crucial part in multiple biological processes of cell proliferation, migration, apoptosis, and chemoresistance. In cancer, miRNAs can be divided into oncogenes or tumor suppressors on the basis of their functions in the carcinogenic process. The purpose of this study was to explore the roles and clinical diagnostic value of miR-370-3p in breast cancer. Our results demonstrated that miR-370-3p significantly promoted proliferation, metastasis, and stemness of breast cancer *in vitro* and *in vivo*. In particular, clinical data revealed that high expression of serum miR-370-3p and exosomal miR-370-3p from breast cancer patients was remarkably correlated with lymphatic metastasis and tumor node metastasis (TNM) stages. Mechanistically, miR-370-3p inhibited FBLN5 expression and activated the NF-*κ*B signaling pathway to promote breast cancer cell proliferation, migration, and stemness. FBLN5 expression was significantly decreased in breast cancer cells and tumor tissues of breast cancer patients. Our research identified that miR-370-3p promoted breast cancer progression by inhibiting FBLN5 expression and activating the NF-*κ*B signaling pathway. Serum exosomal miR-370-3p would provide a potential biomarker for the diagnosis of breast cancer.

## 1. Introduction

Breast cancer is the most common form of cancer among women. Its incidence rate ranks first in malignant tumors of women [1], and it is also the second leading cause of cancer-related death in women [2]. Therefore, it is necessary to find out molecules that affect the progress of breast cancer, then study their functions and mechanisms, and explore their values as novel targets for early detection and treatment of breast cancer.

MicroRNAs (miRNAs) have emerged as important roles in the carcinogenesis and progression of breast cancer by regulating target gene expression *via* posttranscriptional processing [3, 4]. It has been previously reported that miR-128 can target Bcl-2-related x (Bax) gene to promote the proliferation of MCF-7 cells [5]. Metastatic breast cancer cells (MBC) could secrete and transport miR-1246 to mammary epithelial cells and nonmetastatic breast cancer cells to suppress the expression of cyclin-g2, thereby enhancing survival rates and mobility of breast cancer cells [6].

Except for affecting the progression of breast cancer, miRNAs can also be identified as good biomarkers to increase diagnostic accuracy of breast cancer. The combination of miR-21 and miR-1246 is beneficial to the early diagnosis of breast cancer [7, 8]. In addition, serum miR-301 is also considered an early diagnostic marker of breast cancer [9]. miR-101, miR-372, or miR-373 overexpression in serum is also helpful for the diagnosis of breast cancer [10].

In our previous research, we found that bacterial lipopolysaccharide (LPS) promoted the metastasis of breast cancer cells *in vitro* and *in vivo* by activating the TLR4/MyD88/NF-*κ*B signaling pathway. To further determine whether miRNA is involved in this process, we used microarray to screen and found that the breast cancer cells with higher metastasis ability significantly overexpressed miR-370-3p. However, the biological characteristics and molecular mechanisms of miR-370-3p in breast cancer are not clear. This study will clarify the effects of miR-370-3p on the progression of breast cancer and its clinical diagnosis value serving as a novel biomarker.

## 2. Materials and Methods

### 2.1. Cell Culture

Human breast epithelial cell line MCF-10A and human breast cancer cell lines MCF-7 and MDA-MB-231 were purchased from the cell bank of Chinese Academy of Sciences (Shanghai, China). MCF-10A was cultured in RPMI-1640 containing 20% FBS (GIBCO). MCF-7 and MDA-MB-231 were propagated with high-glucose DMEM (GIBCO) containing 10% FBS. 293T cells were preserved in high-glucose DMEM (GIBCO) supplemented with 10% FBS. All cell lines were propagated at 37°C in an incubator containing 5% carbon dioxide.

### 2.2. Human Clinical Specimens

The ethics committee from the Second Affiliated Hospital of Soochow University has approved the study (Project No.: jd-lk-2019-065-01), and the patients involved have known and agreed. The specimens were taken from the cancer tissue and the surrounding normal tissue of female patients with breast cancer in our hospital. The serum was collected from female breast cancer patients in the hospital, and the serum of healthy female physical examinees with similar age was collected. The intravenous blood samples were centrifuged at 4°C at 3000 *g* for 10 minutes. The supernatant was then put into a new centrifuge tube and centrifuged for 10 minutes at 12000 *g* at 4°C. Finally, the remaining serum was stored at -80° C. All specimens were preserved in liquid nitrogen. Meanwhile, the pathological data of breast cancer patients were collected.

### 2.3. Exosomes Isolation and Identification

Exosomes in serum were separated according to the operating instructions in the ExoQuick precipitation solution (System Biosciences, Mountain View, CA, USA). In short, 63 *μ*l ExoQuick solution was added to 250 *μ*l serum and stored in a 4°C refrigerator overnight. The supernatant was centrifuged at 1500 *g* at 4°C for 30 minutes. The supernatant was removed and resuspended with 60 *μ*l PBS.

### 2.4. Transfection

The transient transfection of miR-370-3p was achieved by transfection of miRNA oligonucleotides (miR-370-3p mimics, inhibitor, and corresponding negative control were synthesized by GenePharma, China). According to the instructions, miR-370-3p mimics and inhibitors were, respectively, transfected at 100 nM and 250 nM in breast cancer cells by using Lipofectamine 2000 (Invitrogen, Carlsbad, CA, USA). The target sequences of the oligonucleotides are provided in Supplementary Table S1.

### 2.5. RNA Extraction and Real-Time RT-PCR

TRIzol Reagent (Invitrogen, USA) can be used for total RNA extraction from tissues and cells. Total RNA in serum and exosomes was extracted with QIAzol Lysis Reagent (Invitrogen, USA). The miScript II RT Kit (Qiagen, Germany) can be used to reverse transcribe mRNAs, and the miScript SYBR Green PCR Kit (Qiagen, Germany) can be used to detect them. Similarity, the miScript II RT Kit (Qiagen, Germany) is used for miRNA reverse transcription, and the SYBR Green PCR Kit (Qiagen, Germany) can be used to detect reverse transcriptional mRNA. Quantitative analyses can use CFX96 Real-Time PCR Detection System (Bio-Rad Laboratories, Hercules, CA, USA). All experiments were repeated three times. The internal reference of mRNAs is GAPDH. The internal parameter of miRNAs is U6. Sangon (Shanghai, China) (Supplementary Table S2) designed and synthesized primers for mRNAs. The primers for miRNA detection were purchased from Qiagen.

### 2.6. Double Luciferase Reporter Gene Experiment

The 3′-UTR region of the target gene was constructed and linked to the pmirGLO dual-luciferase miRNA target expression vector (Promega, Madison, WI). Mutation sites were constructed using the Quick Change II Site-Directed Mutagenesis Kit (Agilent Technologies, Santa Clara, CA). In the overexpression system, the reporter vector was transfected into 293T cells together with MNC and 370-mimics. A Dual-Glo luciferase assay system (Promega, Madison, WI) can detect firefly luciferase activity, and the activity was standardized to that of Renilla luciferase. All experiments were repeated three times.

### 2.7. Western Blot

The samples were cleaved with RIPA buffer containing protease inhibitor. 10% SDS-PAGE gel was used to separate the protein extracted from the samples. After electrophoresis, the proteins on the gel were transferred onto 0.22 *μ*m polyvinylidene fluoride membrane and blocked in 5% (*w*/*v*) skimmed milk powder, then incubated with primary antibodies against E-cadherin, N-cadherin, NF-*κ*B-p65 (Cell Signaling Technology, Beverly, MA, USA), vimentin, PCNA (Bioworld Technology, Louis Park, MN, USA), FBLN5 (Santa Cruz Biotechnology, USA); OCT4(Cell Signaling Technology, Beverly, MA, USA), SOX2(Cell Signaling Technology, Beverly, MA, USA), and GAPDH; then, the membrane was fully washed, and the HRP-linked secondary antibodies were used (Kangcheng, Shanghai, China). Finally, the enhanced chemiluminescence system (ImageQuant LAS 4000 mini, GE, Japan) detected the signals. All experiments were repeated three times.

### 2.8. Colony Formation Assay

Transfected cells were plated into 35 mm plates (1000 cells/well) and propagated for 2 weeks. The medium was changed once every three days. Finally, the colonies were fixed with 4% paraformaldehyde and stained with crystal violet. 4% paraformaldehyde fixed the cells, and crystal violet stained them for 20 minutes. PBS was used to wash the dye, and photos were taken under microscope to analyze the results. All experiments were repeated three times.

### 2.9. Transwell Migration Assay

Transfected cells were seeded with appropriate density (1 × 10^5^/well) into the upper chamber of 24-well Transwell plate, and serum-free medium was used. 600 *μ*l medium containing 10% serum was added into the chamber under the culture plate. After 8 hours, the cells transferred to the lower chamber were fixed with 4% paraformaldehyde and stained with crystal violet for 20 min. Photographs were taken under a microscope, and the number of cells migrating in each region was calculated. All experiments were repeated three times.

### 2.10. Subcutaneous Xenograft Model in BALB/c Nude Mice

The research on experimental animals in this paper has been approved by the Ethics Committee of the Second Affiliated Hospital of Soochow University (Project No.: jd-lk-2019-065-01). Female BALB/C nude mice aged 4-6 weeks were harvested from Suzhou Zhaoyan New Drug Research Center Co., Ltd. MDA-MB-231 successfully transfected with MNC, 370-mimics, INC, and 370-inhibitor, respectively, and were injected into the axillary subcutaneous of each group of nude mice (5 × 10^6^ cells, *n* = 6). After injection for 18 days, the mice were sacrificed, and the breast tumors were photographed and weighted. The tumor volume was measured according to the formula 0.5 × *L* × *W*^2^. *L* represents the longitudinal diameter, and *W* represents the latitudinal diameter.

### 2.11. Statistical Analysis

Statistical analysis was carried out by the software GraphPad Prism5. Unpaired-sample *t*-test can be used to analyze the potential expression differences between the two groups after different treatments. The differences of miRNA expression between breast cancer patients and healthy volunteers were analyzed by the Mann–Whitney *U* test. The Wilcoxon signed-rank test was used to analyze the mRNA levels of miR-370-3p and FBLN5 in paired breast cancer tissues and adjacent normal tissues. *P* < 0.05 was considered significant.

## 3. Results

### 3.1. miR-370-3p Stimulates Proliferation and Migration of Breast Cancer Cells

**Figure 1:**
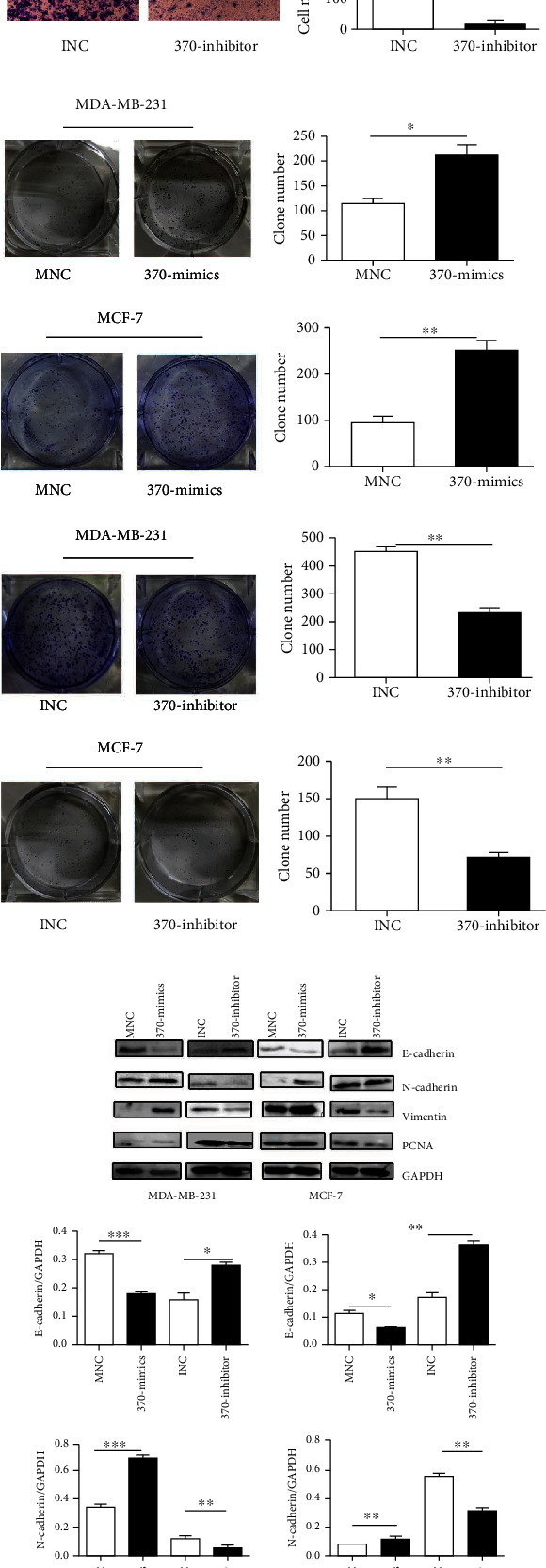
miR-370-3p can promote migration and proliferation of breast cancer cells *in vitro*. (a) Transwell migration assay was used to evaluate the migration of miR-370-3p in breast cancer cells (MDA-MB-231 and MCF-7). ^∗∗∗^*P* < 0.001. The scale bar is in the upper right of the first picture in each row. (b) Evaluation of miR-370-3p on proliferation of breast cancer cells (MDA-MB-231 and MCF-7) by colony forming assay. ^∗∗^*P* < 0.01, ^∗^*P* < 0.05. (c) Western blot was used to detect the expression of EMT-related proteins and PCNA protein of miR-370-3p in breast cancer cells (MDA-MB-231 and MCF-7). ^∗∗∗^*P* < 0.001, ^∗∗^*P* < 0.01, ^∗^*P* < 0.05.

**Figure 2:**
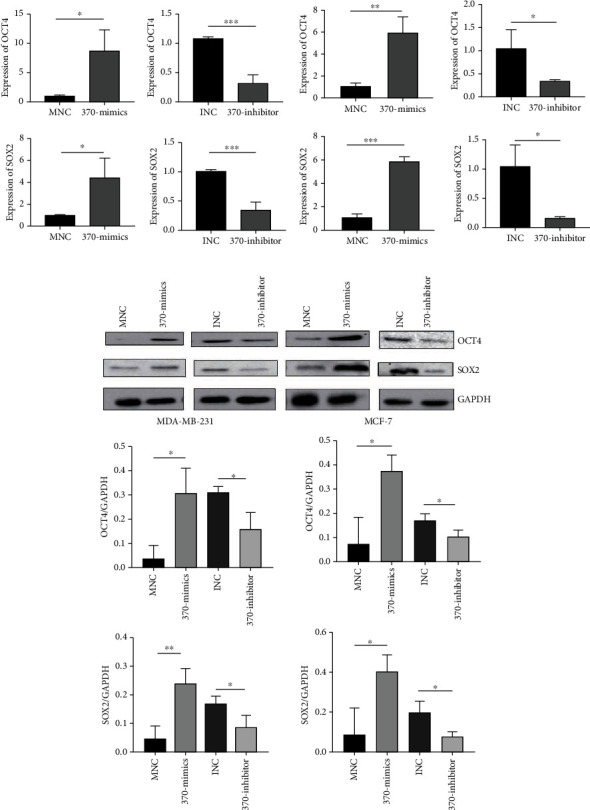
miR-370-3p promotes stemness of breast cancer cells *in vitro*. (a) The expression of OCT4 and SOX2 with MNC, 370-mimics, INC, and 370-inhibitor transfected breast cancer cells were examined by using quantitative RT-PCR. ^∗∗∗^*P* < 0.001, ^∗∗^*P* < 0.01, ^∗^*P* < 0.05. (b) The expression of stemness-related proteins in control and miR-370-3p-overexpressing cells or miR-370-3p knockdown cells was examined by using western blot. ^∗∗^*P* < 0.01, ^∗^*P* < 0.05.

In previous research, we have found that TLR4 was linked to the metastasis of breast cancer *in vitro* and *in vivo* by activating the TLR4/MyD88/NF-*κ*B signaling pathway. The miR-370-3p level was dramatically increased in TLR4-activated breast cancer cells. In the current research, we aimed to study the effect of miR-370-3p in breast cancer. It was found that compared with mammary epithelial cells MCF-10A, miR-370-3p was markedly upregulated in breast cancer cells MCF-7 and MDA-MB-231. Additionally, in MDA-MB-231 cells, the expression of miR-370-3p was higher than that in MCF-7 (S1A), which implied that miR-370-3p might play as an oncomiRNA in the development of breast cancer. For further analysis, we transfected MNC and 370-mimics in MCF-7 and MDA-MB-231. The result of quantitative PCR indicated that the expression levels of miR-370-3p were significantly increased in the transfection group of 370-mimics than that in the control group (S1B). Transwell migration and colony formation assays revealed that miR-370-3p significantly enhanced breast cancer cell migration and proliferation compared to that in the MNC transfection group (Figures 1(a) and 1(b)). The results of western blot showed that the expression of mesenchymal markers N-cadherin, vimentin, and the proliferation-related marker PCNA was increased, whereas the protein levels of epithelial marker E-cadherin was decreased in the 370-mimics transfected cells than that in the control group (Figure 1(c)). Quantitative PCR also showed that the expression levels of stemness-related genes OCT4 and SOX2 were significantly overexpressed in 370-mimics transfection group than in the transfection group of MNC (Figure 2(a)). Western blotting showed that stemness-related proteins OCT4 and SOX2 were significantly higher with the transfection of 370-mimics (Figure 2(b)). Similarly, the expression levels of miR-370-3p were significantly decreased in the 370-inhibitor transfected cells than that in the control group (S1B). The migration and proliferation of breast cancer cells were reduced in 370-inhibitor transfected cells (Figures 1(a) and 1(b)). Moreover, the expression of N-cadherin, vimentin, and PCNA was decreased, whereas the expression of E-cadherin was increased in cells transfected with miR-370-3p inhibitor (Figure 1(c)). The expression levels of OCT4 and SOX2 were also significantly decreased in the transfection group of 370-inhibitor than in the INC transfection group (Figure 2(a)). Western blot results showed that with the transfection of miR-370-3p inhibitor, OCT4 and SOX2 levels were decreased (Figure 2(b)).

**Figure 3:**
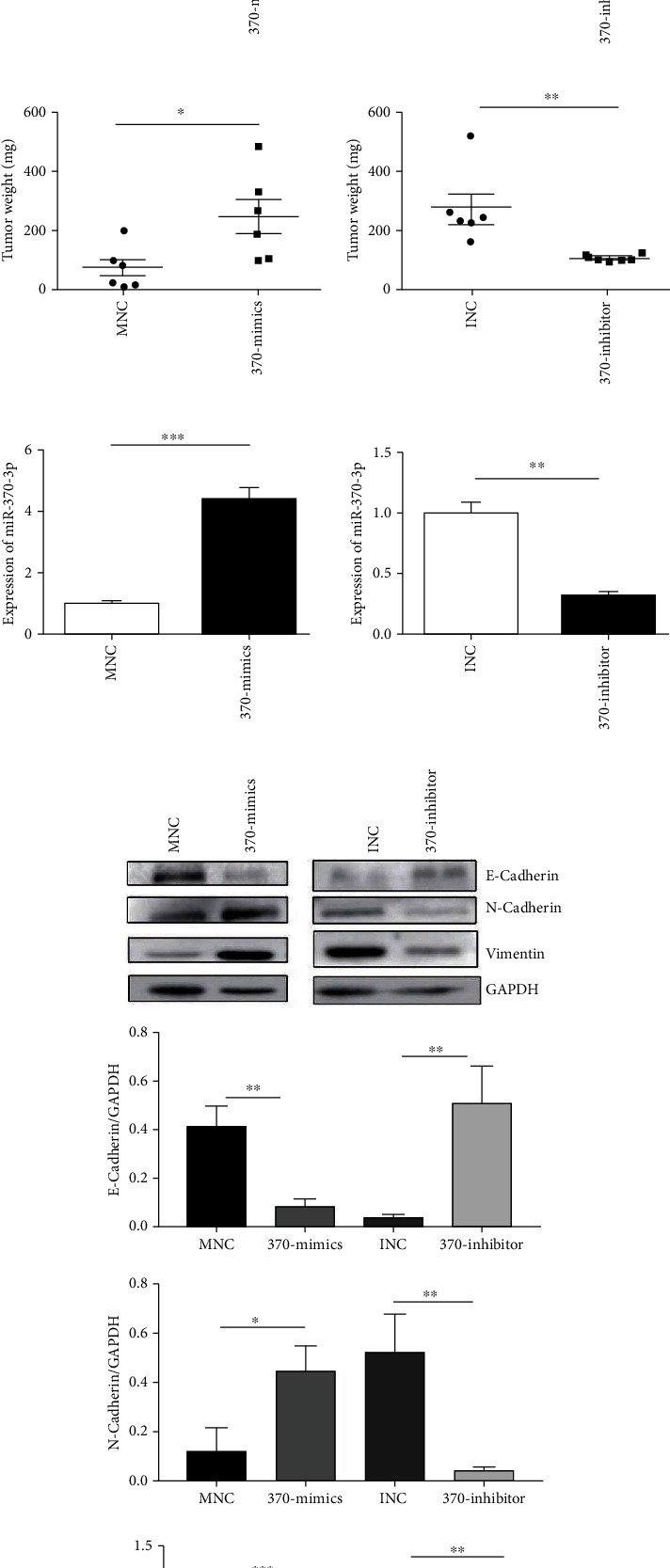
miR-370-3p can promote migration and proliferation of breast cancer cells *in vivo.* (a–c) Observe the appearance, weight, and volume of breast tumor in nude mice subcutaneously formed by MDA-MB-231 cells transfected with MNC, 370-mimics, INC, and 370- inhibitor. ^∗∗^*P* < 0.01, ^∗^*P* < 0.05. (d) The expression of miR-370-3p in the breast tumor tissues formed by MDA-MB-231 cells transfected with MNC, 370-mimics, INC, and 370-inhibitors which were injected into subcutaneous of nude mice was detected by quantitative RT-PCR. ^∗∗^*P* < 0.01, ^∗∗∗^*P* < 0.001. (e) The expression of EMT-related proteins in control and miR-370-3p-overexpressing cells or miR-370-3p knockdown cells were examined by using western blot. ^∗∗∗^*P* < 0.001, ^∗∗^*P* < 0.01, ^∗^*P* < 0.05.

We also researched the significance of miR-370-3p in subcutaneous xenograft models in nude mice. Nude mice were injected into the armpit with breast cancer cell line MDA-MB-231 overexpressed or knocked-down miR-370-3p. The tumor size and weight showed that miR-370-3p mimics treatment remarkably increased tumor growth, and miR-370-3p inhibitor treatment remarkably decreased tumor growth (Figures 3(a)–3(c)). The expression of miR-370-3p was higher in overexpressed miR-370-3p group and lower in knocked-down miR-370-3p group than in the control group (Figure 3(d)). Western blotting showed that the expression of N-cadherin and vimentin related to epithelial-mesenchymal transition (EMT) was significantly higher, but the protein expression of E-cadherin was reduced in the 370-mimics transfection group than in the control group. On the contrary, the results were the opposite (Figure 3(e)). Collectively, the above results demonstrated that miR-370-3p could promote breast cancer growth and metastasis *in vivo*.

### 3.2. MiR-370-3p Promotes Breast Cancer through the Regulation of FBLN5 and the NF-*κ*B Signaling Pathway

**Figure 4:**
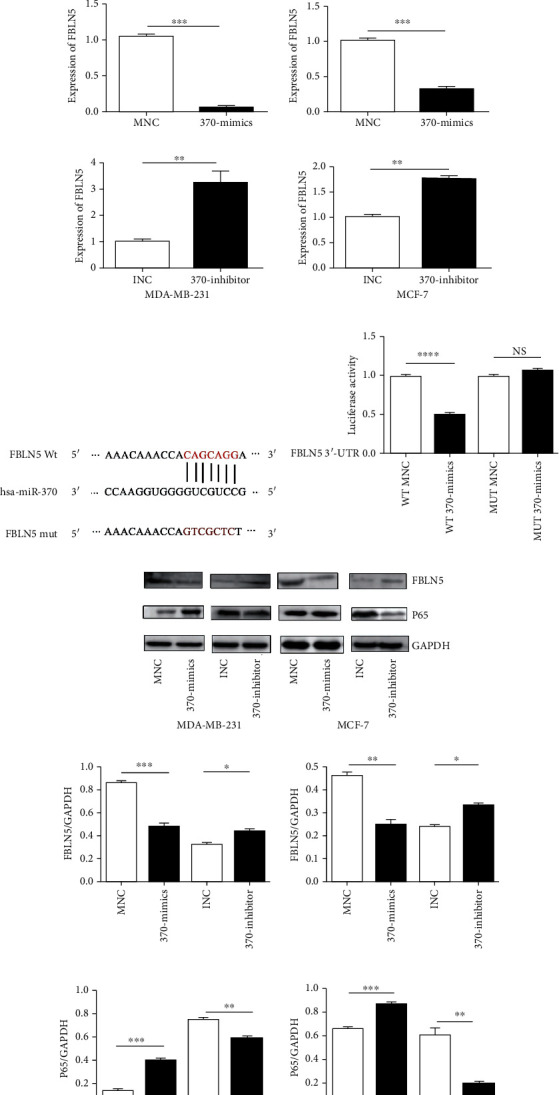
miR-370-3p targets and inhibits FBLN5 and activates the NF-*κ*B signaling pathway. (a) Quantitative RT-PCR was used to detect the expression of FBLN5 in breast cancer cells and them transfected with MNC, 370-mimics, INC, and 370-inhibitor. ^∗∗∗^*P* < 0.001, ^∗∗^*P* < 0.01. (b) The binding sites of miR-370-3p seed region and 3′-UTR of FBLN5 mRNA were predicted. (c) The luciferase reporter gene analysis was used to detect the effect of miR-370-3p on the stability of FBLN5 mRNA. ^∗∗∗^*P* < 0.001. (d) Western blot was used to detect the expression of FBLN5 and p-p65 in breast cancer cells transfected with MNC, 370-mimics, INC, and 370 -inhibitor. ^∗∗∗^*P* < 0.001, ^∗∗^*P* < 0.01, ^∗^*P* < 0.05.

Bioinformatics software miRDB and TargetScan have predicted multiple target genes. The measurement of the expression of predicted target genes in MCF-10A, MCF-7, and MDA-MB-231 and transfected MCF-7 and MDA-MB-231 cells showed a negative correlation between the expression levels of FBLN5 and miR-370-3p, which suggested that FBLN5 might be the downstream gene of miR-370-3p (Figure 4(a)). Wild- and mutant-type reporter gene vectors of the mRNA 3′-UTR binding sites in the target gene FBLN5 were constructed and used to transfect 293T cells together with miR-370-3p mimics. We observed that the luciferase activity of the wild-type reporter gene vector was obviously inhibited by miR-370-3p mimics rather than the mutant reporter gene vector (Figures 4(b) and 4(c)). Furthermore, we found that the expression of FBLN5 was lower in the 370-mimics transfection group and higher in the 370-inhibitor transfection group than in the control group (Figure 4(d)).

It has been reported that FBLN5 can inhibit the NF-*κ*B signaling pathway and participate in fibroblast apoptosis [11]. Our data showed that NF-*κ*B-p65 expression was increased in breast cancer cells overexpressing miR-370-3p while the expression was decreased in cells with knocked-down miR-370-3p (Figure 4(d)). These results suggested that miR-370-3p promoted the proliferation and metastasis of breast cancer cells through directly targeting FBLN5 and regulating the NF-*κ*B signaling pathway.

#### 3.2.1. Clinical Tests and Analysis of FBLN5 in Breast Tissues

**Figure 5:**
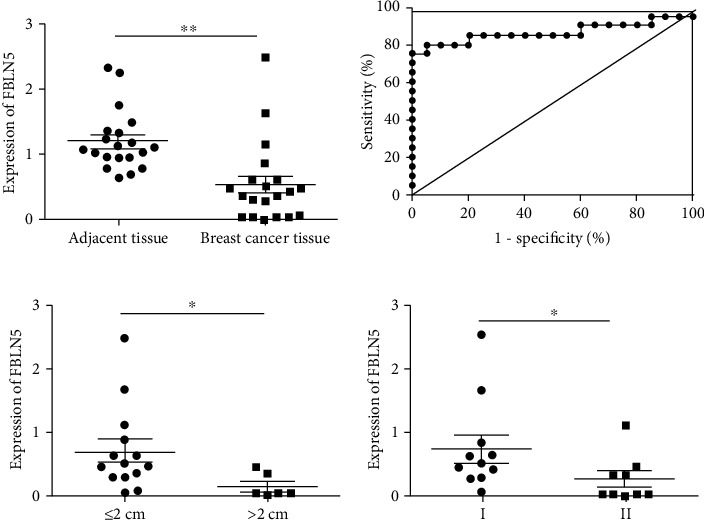
The detection of FBLN5 expression in breast tissues and its clinical correlation analysis. (a) The mRNA levels of FBLN5 in normal tissues adjacent to carcinoma and breast cancer tissues. ^∗∗^*P* < 0.01. (b) The diagnostic values of FBLN5 ROC curves in breast cancer tissues. (c) Correlation between the expression of FBLN5 and clinicopathological parameters (tumor diameter and TNM stage) in breast cancer tissues. ^∗^*P* < 0.05.

We detected the expression levels of FBLN5 in clinical tissue specimens of patients with breast cancer. The level of miR-370-3p was significantly downregulated in 20 breast cancer tissue samples compared with matched adjacent normal tissues (Figure 5(a)). The diagnostic sensitivity, specificity, and area under the receiver operating characteristic (ROC) curve for FBLLN5 in tissues were 80%, 95%, and 0.8650, respectively (Figure 5(b)). FBLN5 may serve as a novel molecular marker in tissue detection for breast cancer.

Combined with the clinicopathological data, we found that FBLN5 was correlated with clinicopathological parameters in breast cancer tissues (Table 1). The expression levels of FBLN5 in breast cancer patients were negatively related with tumor diameter and TNM stage (Figure 5(c)). Taken together, these findings suggested that FBLN5 inhibited the development of breast cancer.

### 3.3. miR-370-3p Is Highly Expressed in Breast Tissues, Serum, and Serum Exosomes

**Figure 6:**
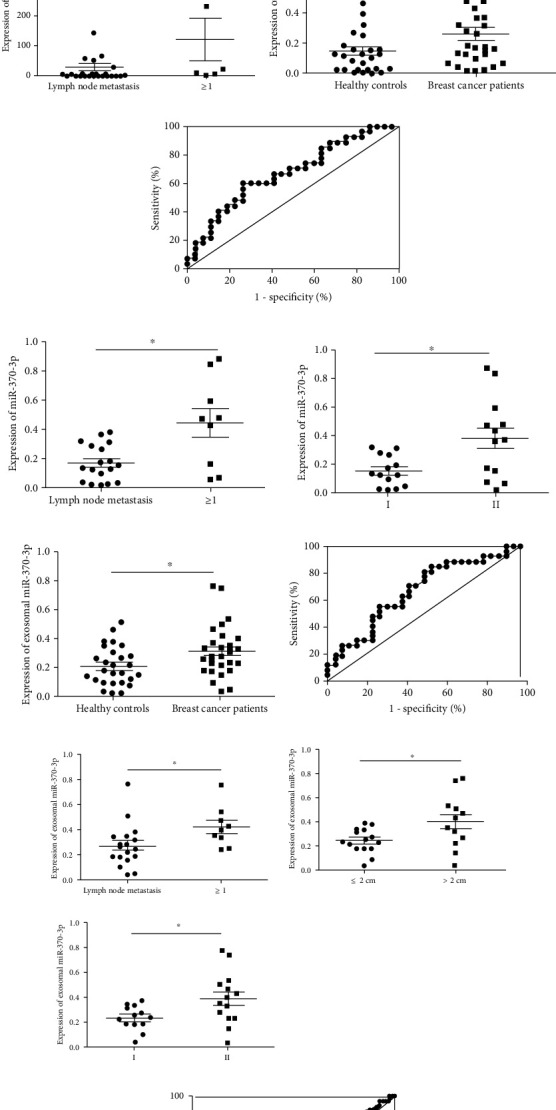
The detection of miR-370-3p expression in breast tissues, serum, and serum exosomes. (a) The levels of miR-370-3p in adjacent normal tissues and breast cancer tissues. ^∗∗∗^*P* < 0.001. (b) The diagnostic values of miR-370-3p ROC curves in breast cancer tissues. (c) The correlation between the levels of miR-370-3p and clinicopathological parameters (lymph node metastasis) in breast cancer tissues. ^∗^*P* < 0.05. (d) The expression of miR-370-3p in the serum of 28 healthy volunteers and that of 28 patients with breast cancer. ^∗^*P* < 0.05. (e) The diagnostic values of miR-370-3p ROC curves in breast cancer serum. (f) The correlation between the levels of miR-370-3p and clinicopathological parameters (lymph node metastasis and TNM stage) in breast cancer serum. (g) The levels of miR-370-3p in the exosomes of serum in 28 healthy volunteers and that of 28 patients with breast cancer. ^∗^*P* < 0.05. (h) The diagnostic values of miR-370-3p ROC curves in breast cancer serum exosomes. (i) The correlation between the levels of miR-370-3p and clinicopathological parameters (lymph node metastasis, tumor diameter, and TNM stage) in serum exosomes of breast cancer. ^∗^*P* < 0.05. (j) The ROC curve of serum CA153.

To extend current knowledge to clinical patients, we first detected the expression of miR-370-3p in 31 paired breast cancer tissue specimens and adjacent tissue specimens. The results showed that the expression of miR-370-3p in 31 breast cancer tissue specimens exhibited significant increase compared to the paired adjacent tissue specimens (Figure 6(a)). The diagnostic sensitivity, specificity, and area under the ROC curve for miR-370-3p in tissue were 74.19%, 96.77%, and 0.7534, respectively (Figure 6(b)). The expression levels of miR-370-3p were also elevated in clinical serum and exosomes specimens (Figures 6(d) and 6(g)). The expression of miR-370-3p was remarkably upregulated in both samples from breast cancer patients compared with healthy persons. The diagnostic sensitivity, specificity, and area under the ROC curve for miR-370-3p in serum and exosomes were 59.26%, 74.07%, and 0.6735 (Figure 6(e)) and 55.56%, 74.07%, and 0.6797 (Figure 6(h)), respectively. Moreover, our results also showed that diagnostic sensitivity, specificity, and area under the ROC curve of CA153 were 49.18%, 82.54%, and 0.6177 (Figure 6(j)).

Combined with the clinicopathological data, the correlations between the expression of miR-370-3p and clinicopathological parameters in breast cancer tissues, serum, and exosomes were analyzed (Tables 2–4). The expression levels of miR-370-3p in breast cancer patients with lymph node metastasis were higher than those without lymph node metastasis (Figure 6(c)). The expression of miR-370-3p in the lymph node metastasis group and TNM II group was significantly higher than that in breast cancer non-lymph node metastasis and the TNM I group. (Figure 6(f)). The expression of serum exosomal miR-370-3p in tumor diameter > 2 cm, lymph node metastasis, and TNM stage II group were significantly higher than that in breast cancer tumor diameter ≤ 2 cm, non-lymph node metastasis, and TNM stage I group (Figure 6(i)).

## 4. Discussion

Breast cancer is a complex malignant disease involving tumor formation, proliferation, metastasis, invasion, angiogenesis, recurrence, and other pathological processes [12]. Therefore, it is essential to further investigate the molecular functions and mechanisms in the development of breast cancer.

In this study, we first demonstrated roles of miR-370-3p in breast cancer cells. We found that overexpressed miR-370-3p *in vivo* promoted the proliferation, mobility, and stemness of breast cancer cells, whereas miR-370-3p knockdown significantly offsets these abilities of breast cancer cells, indicating an oncomiRNA in breast cancer. At the same time, we also explored the effects of miR-370-3p on tumor cells of nude mice, which showed that miR-370-3p promoted the proliferation and migration *in vivo.*

We further investigated the molecular mechanism of miR-370-3p in promoting the proliferation, metastasis, and stemness of breast cancer cells. We finally confirmed that FBLN5 is a target gene directly regulated by miR-370-3p. It has been reported that miR-200c is overexpressed in uterine leiomyoma by targeting FBLN5 [13]. Fibulin-5 is expressed lowly in colorectal cancer and correlated with clinicopathologic characteristics [14]. Endometrial miR-200c is altered during the transformation into cancerous states and targets FBLN5 [15]. In our study, we found that the expression of FBLN5 in breast cancer cells and breast cancer tissues was lower than that in breast epithelial cells and normal breast tissues, which confirmed that the expression level of FBLN5 was negatively correlated with miR-370-3p. These findings suggested that miR-370-3p promoted the proliferation, migration, and stemness of breast cancer cells, which was related to the underexpressed FBLN5. NF-*κ*B is involved in the inflammatory and immune response and regulates cell apoptosis and stress response. The abnormal expression of NF-*κ*B is closely related to the occurrence and development of cancer [16, 17]. It has been reported that KRAS induced lung adenocarcinoma can activate the NF-*κ*B pathway and promote tumor proliferation [18–20]. NF-*κ*B plays a key role in the proliferation, apoptosis, angiogenesis, and metastasis of colorectal cancer cells [21]. We found that miR-370-3p activated the NF-*κ*B signaling pathway by targeting FBLN5 to promote the proliferation, migration, and stemness of breast cancer cells.

The downregulated expression of FBLN5 in breast cancer suggested that FBLN5 played the role of tumor suppressor gene in breast cancer. FBLN5 is a tumor suppressor that can inhibit the migration and invasion of ovarian cancer cells [22]. Therefore, we studied the expression level of FBLN5 in clinical breast cancer and breast cancer tissues, as well as its correlations with clinicopathological parameters. The results showed that the expression of FBLN5 in breast cancer was lower than that in the adjacent tissues, and the area under the ROC curve was 0.8650, which had better diagnostic efficiency. The sensitivity and specificity of the diagnosis were 80% and 95%, respectively, which indicated that FBLN5 could be a potential candidate of tissue detection for breast cancer diagnosis. In addition, FBLN5 was negatively correlated with tumor diameter and TNM stage of breast cancer, suggesting that low expression of FBLN5 was a predictor of poor prognosis.

The expression level of miRNA is closely related to the morphological characteristics, histopathological parameters, and clinical diagnosis of breast cancer [23]. There are few reports about miR-370-3p in breast cancer. We first measured the expression of miR-370-3p in breast cancer tissues and adjacent tissues. The results showed that the expression of miR-370-3p in breast cancer tissues was significantly higher than that in adjacent tissues, and the ROC curve showed that miR-370-3p had better diagnostic efficiency and higher sensitivity and specificity in breast cancer tissues.

The detection of miR-370-3p in tissues is invasive. Although histopathological detection is the gold standard for breast cancer detection, it is difficult to obtain samples, and the detection cycle is too long to make a timely diagnosis of patients' disease status. miR-370-3p in circulating fluid such as serum and exosomes which come from tissues can well reflect the patient's condition and is easy to obtain and detect.

The results showed that the expression of miR-370-3p in the serum of breast cancer patients was significantly higher than that of healthy people. CA153 is one of the most important specific markers of breast cancer, and we also detected the expression of CA153 in the serum. miR-370-3p in serum had certain diagnostic efficiency that was better than CA153 and higher detection specificity, which can be used as a biological marker for breast cancer diagnosis. Combined with clinicopathological data, we found that the expression level of miR-370-3p was positively correlated with lymph node metastasis and TNM stage.

Exosomes contain many kinds of nucleic acids, especially miRNAs. These miRNAs are selectively sorted into exosomes of different origins [24, 25]. In recent years, many literatures have reported the clinical application of miRNAs in exosomes of breast cancer. Studies have shown that the levels of miR-376a, miR-27a, miR-155, and miR-376c from serum exosomes dynamically reflect the disease status of breast cancer patients [26]. In this study, we identified an increased expression of miR-370-3p in serum exosomes of breast cancer than in healthy people, suggesting that exosomal miR-370-3p may be a new marker for diagnosis and prognosis of breast cancer. Serum exosomal miR-370-3p had certain diagnostic efficacy and higher detection specificity, which can be acted as a biological index for the diagnosis of breast cancer. The diagnostic efficiency of serum miR-370-3p and serum exosomal miR-370-3p was better than CA153, which means serum and exosomal miR-370-3p may be a potential biomarker. Combined with clinical-pathological data, the results indicated that the expression of miR-370-3p in exosomes was positively correlated with tumor diameter, lymph node metastasis, and TNM stage. Taken together, miR-370-3p was upregulated in different kinds of specimens and promoted breast cancer progression vigorously.

## 5. Conclusions

Collectively, our data firstly demonstrated that miR-370-3p acted as an oncogene in the development of breast cancer by inhibiting FBLN5 expression and activating the NF-*κ*B signaling pathway. This study will offer a new insight into roles of miR-370-3p in breast cancer progression and provide a novel biomarker for the diagnosis and therapy of breast cancer.

## Figures and Tables

**Table 1:** Correlation between FBLN5 in breast cancer tissues and clinicopathological parameters.

Clinicopathological information	n	Relative expression of FBLN5	*P*
Tumor diameter			
<=2cm	14	0.481(0.284, 0.915)	0.015
>2cm	6	0.029(0.020, 0.366)	
Age			
<=50	8	0.630(0.273, 1.049)	0.0836
>50	12	0.312(0.029, 0.452)	
Pathological type			
Ductal carcinoma in situ	3	0.338(0.028, 2.489)	0.4472
Invasive ductal carcinoma	14	0.316(0.029, 0.588)	
Others	3	0.624(0.426, 0.636)	
Lymph node metastasis			
0	16	0.439(0.222, 0.627)	0.3694
>=1	4	0.187(0.021, 0.542)	
TNM staging			
I	11	0.503(0.286, 0.844)	0.0227
II	9	0.029(0.028, 0.372)	
ER			
No data	3	0.503(0.053, 1.663)	
-	5	0.028(0.020, 0.643)	0.0651
+	12	0.439 (0.325, 0.627)	
PR			
No data	3	0.503(0.053, 1.663)	
-	5	0.028 (0.020, 0.960)	0.2684
+	12	0.386 (0.284, 0.627)	
HER-2			
No data	4	0.278 (0.047, 0.793)	
+	2	0.435 (0.020, 0.633)	0.6791
++	11	0.451 (0.279, 0.636)	
+++	3	0.338 (0.001, 0.426)	

**Table 2:** Correlation between miR-370-3p in breast cancer tissues and clinicopathological parameters.

Clinicopathological information	n	Relative expression of miR-370-3p	*P*
Tumor diameter			
<=2cm	18	6.371(1.450, 58.84)	0.1864
>2cm	13	2.389(0.696, 12.60)	
Age			
<=50	8	2.383(1.432, 16.83)	0.4701
>50	23	6.951(1.428, 28.49)	
Pathological type			
Ductal carcinoma in situ	3	1.428(0.068, 28.49)	0.5331
Invasive ductal carcinoma	18	5.507(1.500, 87.45)	
Mucinous adenocarcinoma	2	2.781(1.033, 3.139)	
Others	8	6.549(1.405, 31.13)	
Lymph node metastasis			
0	25	3.506(1.350, 14.18)	0.0483
>=1	6	18.32(6.029, 285.1)	
TNM staging			
I	14	2.781(1.350, 20.94)	0.5515
II	17	6.951(1.249, 33.85)	
ER			
-	10	1.439(0.067, 87.44)	0.1631
+	21	6.951 (2.167, 25.76)	
PR			
-	10	2.478 (0.067, 166.8)	0.6420
+	21	5.792 (1.500, 15.05)	
HER-2			
-	3	0.711 (0.062, 1.500)	0.6175
+	6	0.541 (1.339, 32.54)	
++	15	5.792 (2.389, 11.78)	
+++	6	39.21 (0.968, 87.44)	

**Table 3:** Correlation between miR-370-3p in breast cancer serum and clinicopathological parameters.

Clinicopathological information	n	Relative expression of miR-370-3p	*P*
Tumor diameter			
<=2cm	16	0.136(0.058, 0.268)	0.0513
>2cm	11	0.377(0.161, 0.481)	
Age			
<=50	7	0.130(0.054, 0.197)	0.1530
>50	20	0.264(0.093, 0.444)	
Pathological type			
Ductal carcinoma in situ	2	0.079(0.027, 0.102)	0.6287
Invasive ductal carcinoma	9	0.281(0.138, 0.348)	
Invasive cancer	13	0.168(0.091, 0.327)	
Others	3	0.364(0.264, 0.481)	
Lymph node metastasis			
0	18	0.149(0.040, 0.281)	0.0168
>=1	9	0.480(0.143, 0.651)	
TNM staging			
I	14	0.136(0.040, 0.268)	0.0308
II	13	0.377(0.138, 0.508)	

**Table 4:** The relationship between miR-370-3p in exosomes of breast cancer serum and clinicopathological parameters.

Clinicopathological information	n	Relative expression of miR-370-3p	*P*
Tumor diameter			
<=2cm	15	0.239(0.184, 0.337)	0.0429
>2cm	12	0.389(0.268, 0.514)	
Age			
<=50	7	0.279(0.182, 0.344)	0.3911
>50	20	0.324(0.211, 0.437)	
Pathological type			
Ductal carcinoma in situ	2	0.553(0.258, 0.572)	0.1583
Invasive ductal carcinoma	8	0.379(0.210, 0.478)	
Invasive cancer	14	0.248(0.162, 0.321)	
Others	3	0.351(0.153, 0.374)	
Lymph node metastasis			
0	18	0.247(0.174, 0.339)	0.0193
>=1	9	0.401(0.308, 0.485)	
TNM staging			
I	13	0.237(0.184, 0.322)	0.0273
II	14	0.376(0.238, 0.513)	

## Data Availability

The data used to support the findings of this study are included within the article.

## Supplementary Materials

Supplementary Materials Table S1: sequences of oligonucleotide fragment and modification. Table S2: primer sequences and amplified fragment products.
