# Supplementary material for: miR-370-3p as a Novel Biomarker Promotes Breast Cancer Progression by Targeting FBLN5
Source: Stem Cells Int. 2021 Aug 23;2021:4649890. doi: 10.1155/2021/4649890 (PMC8407987; doi:10.1155/2021/4649890)
Supplement: Supplementary Materials — Table S1: sequences of oligonucleotide fragment and modification. Table S2: primer sequences and amplified fragment products. [file 4649890.f1.zip › Supplementary Table S1.docx]

Table S1 Sequences of oligonucleotide fragment and modification

| **Oligonucleotides** | **sequence(5’-3’)** | **Modification** |
| --- | --- | --- |
| Mimics negative control | Sense:UUCUCCGAACGUGUCACGUTT | 2’Ome |
|  | Antisense:ACGUGACACGUUCGGAGAATT |  |
| miR-370-3p mimics | Sense:GCCUGCUGGGGUGGAACCUGGU | 2’Ome |
|  | Antisense:CAGGUUCCACCCCAGCAGGCUU |  |
| Inhibitor negative control | CAGUACUUUUGUGUAGUACAA | 2’Ome |
| miR-370-3p inhibitor | ACCAGGUUCCACCCCAGCAGGC | 2’Ome |

Note: 2’Ome, 2’-O-Methyl-modified.
