# Supplementary figures and images for: miR-370-3p as a Novel Biomarker Promotes Breast Cancer Progression by Targeting FBLN5
Source: Stem Cells Int. 2021 Aug 23;2021:4649890. doi: 10.1155/2021/4649890 (PMC8407987; doi:10.1155/2021/4649890)

A

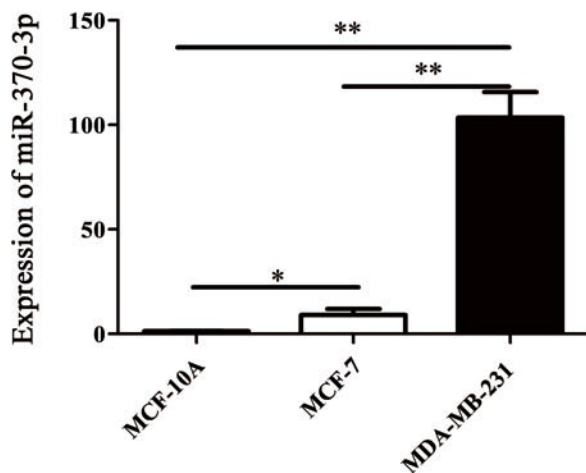

B

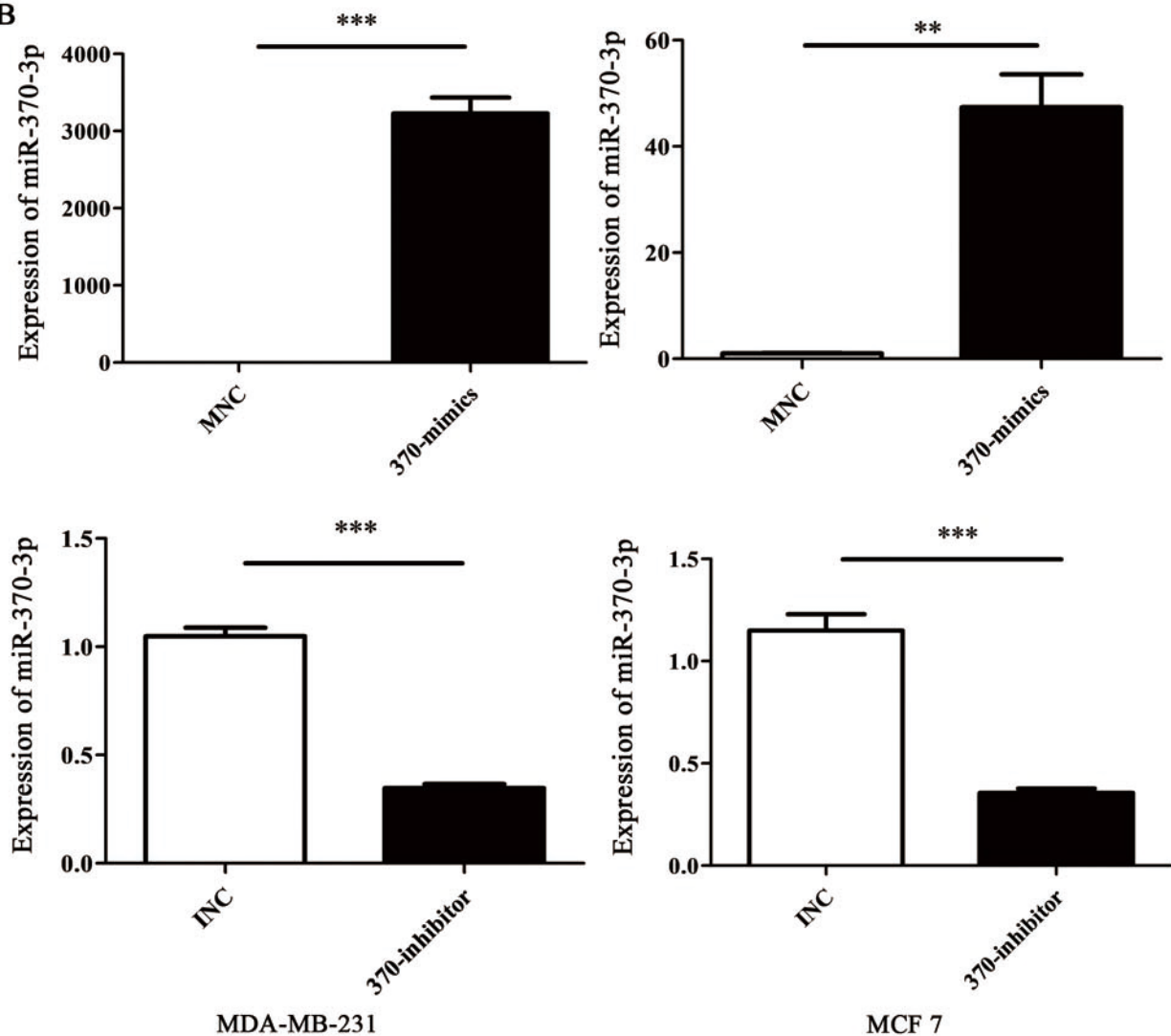

Supplement: Supplementary Materials — Table S1: sequences of oligonucleotide fragment and modification. Table S2: primer sequences and amplified fragment products. [file 4649890.f1.zip › Supplementary Figure S1.pdf]
