# Supplementary material for: miR-370-3p as a Novel Biomarker Promotes Breast Cancer Progression by Targeting FBLN5
Source: Stem Cells Int. 2021 Aug 23;2021:4649890. doi: 10.1155/2021/4649890 (PMC8407987; doi:10.1155/2021/4649890)
Supplement: Supplementary Materials — Table S1: sequences of oligonucleotide fragment and modification. Table S2: primer sequences and amplified fragment products. [file 4649890.f1.zip › Supplementary Table S2.doc]

Table S2 Primer sequences and amplified fragment products

| Genes | Primer sequences（5’- 3’） |
| --- | --- |
| FBLN5 | For: CTGTGACCCAGGATATGAACTT  Rev: TTGTAAATTGTAGCACGTCTGC |
| Oct4 | For：TGGAGAAGGTGGAACCAACT Rev：AGATGGTGGTCTGGCTGAAC |
| Sox2 | For：TTGAGGCTCTGCAGCTTAG  Rev：GCCGGTTACAGAACCACAC |
| GAPDH | For: GGGAAACTGTGGCGTGAT  Rev: GAGTGGGTGTCGCTGTTGA |
